# Cardiac sympathetic nerve activity following mitral transcatheter edge-to-edge repair verified by ^123^I-metaiodobezylguanidine (MIBG) scintigraphy among disproportionate secondary mitral regurgitation

**DOI:** 10.1007/s00380-026-02661-8

**Published:** 2026-02-23

**Authors:** Takahide Arai, Yodo Gatate, Keisuke Matsuo, Mitsunobu Nagai, Hiroki Hoya, Yohji Matsusaka, Yoshie Nakajima, Shiro Iwanaga, Ichiei Kuji, Shintaro Nakano

**Affiliations:** 1 Department of Cardiology, Saitama Medical University, International Medical Center, 1397-1 Yamane, Hidaka-Shi, Saitama, Japan; 2 Department of Nuclear Medicine, Saitama Medical University, International Medical Center, Saitama, Japan

**Keywords:** Mitral regurgitation, Transcatheter edge-to-edge repair, ^123^I-metaiodobezylguanidine (MIBG) scintigraphy, Proportionality, Cardiac sympathetic nerve

## Abstract

This study evaluated the cardiac sympathetic nerve (CSN) activity following mitral transcatheter edge-to-edge repair (M-TEER) using ^123^I-metaiodobenzylguanidine (MIBG) scintigraphy among disproportionate secondary mitral regurgitation (SMR). Among the 31 patients who underwent M-TEER between November 2018 and December 2020, 20 patients who underwent pre-procedural and 1-year post-procedural MIBG scintigraphy were included. The ratio of the pre-procedural effective regurgitant orifice area (EROA) to the left ventricular end-diastolic volume (LVEDV) was retrospectively assessed. Patients with higher EROA/LVEDV ratios were classified as MR-dominant/MR-LV-co-dominant (MD/MLCD), indicating disproportionate SMR. The changes of MIBG scintigraphy parameters were assessed among disproportionate SMR patients. All patients presented with heart failure due to moderate-to-severe (3+) or severe (4+) SMR. Following M-TEER, all patients achieved an MR grade of ≤ 2+. Of the 20 patients, 17 (85%) were classified as MD/MLCD, representing disproportionate MR. In terms of MIBG parameters, early and delayed heart-to-mediastinum ratio (e-H/M, d-H/M) showed no significant changes, but washout rate (WR) significantly decreased post-M-TEER (e-H/M; 2.07 ± 0.39 vs. 2.01 ± 0.43, P = 0.35, d-H/M; 1.86 ± 0.41 vs. 1.87 ± 0.44, P = 0.96, WR; 38.8 ± 17.5 % vs. 32.1 ± 15.2 %, P = 0.01). The brain natriuretic peptide level was significantly decreased post-M-TEER (476.7 ± 387.1 pg/mL to 306.7 ± 309.2 pg/mL, P < 0.01). The 5-year mortality prediction model showed no significant difference post-TEER (25.9 ± 15.7 % vs. 20.4 ± 11.3 %, P = 0.45). This study found a significant reduction in CSN tone, evidenced by a reduction in WR, among disproportionate SMR patients who underwent M-TEER. Further research is warranted to elucidate the association between reduced CSN tone and long-term outcomes after M-TEER.

## Introduction

Dysfunction of cardiac sympathetic nerve (CSN) activity has been associated with adverse outcomes in patients with heart failure [1]. Cardiac 123I-metaiodobenzylguanidine (MIBG) scintigraphy is utilized for assessing CSN activity [2]. Previous studies reported that reduced heart-to-mediastinum ratio (H/M) and elevated washout rate (WR) are associated with poorer prognosis in heart failure cohorts [3] and MIBG imaging is recommended for risk stratification in this population [4].

Mitral transcatheter edge-to-edge repair (M-TEER) using the MitraClip® (Abbott Structural Heart, Santa Clara, California) in patients with secondary mitral regurgitation (SMR) has demonstrated safety and superior outcomes compared to medical therapy alone [5]. M-TEER has been shown to improve CSN activity [6]. 

Of note, the concept of proportionality, defined by the effective regurgitant orifice area (EROA) to left ventricular end-diastolic volume (LVEDV) ratio, has been proposed as a novel prognostic indicator following M-TEER [7]. The higher EROA/LVEDV ratio was classified as MR-dominant /MR-LV-co-dominant (MD/MLCD), which indicates a disproportionate SMR. Patients with a MR-dominant/MR-LV-co-dominant (MD/MLCD) had a comparable, adjusted 2-year mortality rate after M-TEER which was slightly better than that of LV-dominant (LD) patients [8].

However, data on the changes in CSN activity following M-TEER among disproportionate SMR are limited. Therefore, this study aimed to investigate the changes in CSN activity following M-TEER among disproportionate SMR, as verified by MIBG scintigraphy.

## Materials and methods 

### Participant eligibility

From November 2018 to December 2020, a retrospective screening was conducted on 31 consecutive patients who underwent M-TEER for moderate to severe or severe MR at our institution. Among these, 20 patients who underwent pre-procedural and 1-year post-procedural MIBG scintigraphy were included. This study was approved by the Clinical Research Appropriate Promotion Center at Saitama Medical University International Medical Center (institutional-ID: 2023-133).

### Data collected at baseline and echocardiographic findings 

Baseline characteristics, including age, sex, comorbidities (hypertension, dyslipidemia, diabetes mellitus, and chronic kidney disease), presence of atrial fibrillation, history of myocardial infarction, coronary artery bypass grafting, percutaneous coronary intervention, and medications, were collected. Brain natriuretic peptide (BNP) levels were recorded before and after M-TEER. Transthoracic and transesophageal echocardiography evaluated MR, EROA, regurgitant volume via the proximal isovelocity surface area (PISA) method, as well as LVEDV, and LV ejection fraction (EF) using Simpson’s method. Patients with higher EROA/LVEDV ratios (≥ 0.165 cm2 per 100 ml of LVEDV) were classified as MR-dominant (MD), intermediate (< 0.165 and ≥ 0.115 cm2 per 100 ml of LVEDV) as MR-LV-co-dominant (MLCD), and low (< 0.115 cm2 per 100 ml of LVEDV) as LV-dominant (LD) as previously described [8].

### M-TEER procedure

M-TEER was performed using the MitraClip® system as previously described [9]. The procedure involved right femoral vein access under general anesthesia and was guided by transesophageal echocardiography. Clips were placed to reduce MR, with additional clips added as needed.

### ^123^I-metaiodobezylguanidine (MIBG) scintigraphy 

Anterior planar and single photon emission computed tomography (SPECT) images were acquired 15 minutes (early) and 3 hours (delayed) after intravenous injection of 111 MBq (3 mCi) of MIBG (PDR Pharma Inc., Tokyo, Japan), with the patient at rest, using the single photon emission computed tomography (SPECT/CT) system (Symbia Intevo 16, Siemens Healthineer, Erlangen, Germany) equipped with a low-energy high-resolution parallel collimator. The acquired planar image was imported to the workstation, and a commercially available software package smartMIBG^Ⓡ^ (PDR Pharma Inc., Tokyo, Japan) was used to perform semi-automatic calculations by standardizing institutional variation [10, 11]. The early and delayed early heart-to-mediastinum ratios (e- and d-H/M) as an institutional value obtained from a low-energy collimator, and WR with decay and background correction were calculated using smartMIBG^Ⓡ^.The WR was calculated from early and delayed planar images. The MIBG protocol was previously described in detail [12]. Delta-WR was computed as follow-up WR minus baseline WR. The-5-year predicted mortality risk was automatically calculated using smartMIBG^Ⓡ^ by adding clinical information including age, sex, and NYHA class to MIBG parameters [13, 14].

### Statistical analysis 

All statistical analyses were performed using SPSS version 21.0 (SPSS, Inc., Chicago, IL, USA). Continuous variables were expressed as mean ± SD or interquartile range (IQR), while dichotomous variables were expressed as numbers and percentages. 95% confidence intervals for key variables were also reported. Measurements were compared using the Wilcoxon signed-rank test. Correlation between WR and BNP reduction was assessed via Pearson analysis. A P-value of < 0.05 was considered statistically significant.

## Results

### Baseline, procedural, postprocedural characteristics

**Fig. 1:**
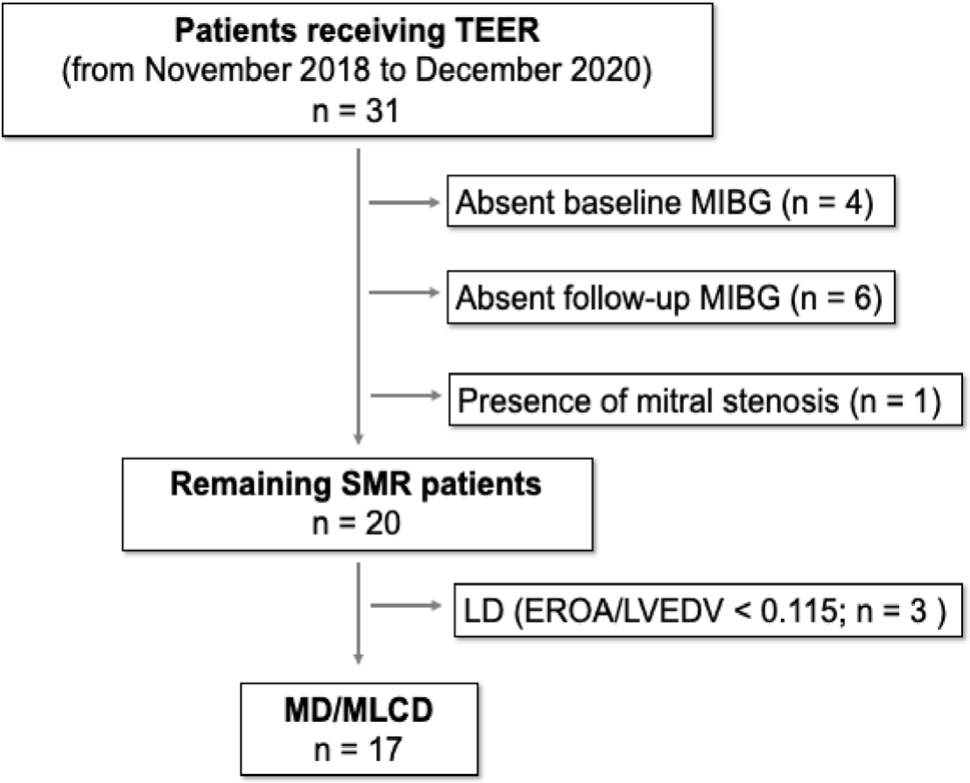
Study eligibility flowchart

Of the 31 patients who underwent M-TEER, those without baseline MIBG (n = 4) and those lacking follow-up MIBG at 1-year post-M-TEER (n = 6) were excluded. The reasons for missing baseline MIBG include patient refusal (n = 2) and scheduling issues (n = 2). While reasons for missing follow-up MIBG included patient refusal (n = 2) and scheduling issues (n = 4). A patient with the presence of mitral stenosis following M-TEER was also excluded (n = 1). The remaining 20 patients were included in the study. Among these, seventeen (85%) were classified as MD/MLCD (Fig. 1). Table 1 summarizes baseline and procedural characteristics and echocardiographic findings. Table 2 summarizes 1-year post-procedural characteristics and echocardiographic findings. No patients underwent additional invasive interventions or clinically significant medication optimization that would be expected to modify MIBG parameters during follow-up (e.g., cardiac resynchronization therapy)*.*

**Table 1:** Baseline, procedural and postprocedural patient characteristics

	MD/MLCD
Patients, n	*n* = 17
Characteristics	
Age, years	71.7 ± 11.5
Male, n	10 (58.8)
Body mass index, kg/m^2^	22.5 ± 1.9
Prior MI, n	5 (29.4)
Prior PCI, n	4 (23.5)
Prior CABG, n	1 (5.9)
Dyslipidemia, n	8 (47.1)
Diabetes mellitus, n	4 (23.5)
Hypertension, n	9 (52.9)
CKD, n	10 (58.8)
Atrial fibrillation, n	8 (47.1)
Cardiac resynchronization therapy, n	6 (35.2)
STS score, %	7.1 ± 7.4
Laboratory data	
BNP, pg/mL	476.7 ± 387.1
eGFR, mL/min/1.73 m^2^	45.1 ± 16.9
Hemoglobin, mg/dL	12.5 ± 2.1
Medications	
ACEi/ARB/ARNI, n	9 (52.9)
βblocker, n	15 (88.2)
MRA, n	11 (64.7)
Echocardiographic data	
MR grade, n	
III / IV	18 (100.0)
LVEF, %	33.9 ± 8.2
LVEDV, mL	173.2 ± 30.7
LVDd, mm	63.1 ± 5.9
LVDs, mm	50.1 ± 7.5
LAD, mm	50.1 ± 7.3
EROA (PISA), cm^2^	0.33 ± 0.12
Procedural variables	
Clip number	
Implantation of one clip, n	11 (64.7)
Implantation of two clip, n	6 (35.3)
Implantation of three clip, n	0 (0)
Single leaflet device attachment, n	0 (0)
Device embolization, n	0 (0)
Any device-related complication requiring cardiovascular surgery, n	0 (0)
Cardiac tamponade, n	0 (0)
Postprocedural variables	
Progressive heart failure, n	0 (0)
In hospital mortality, n	0 (0)

Values are expressed as number (%) or mean ± standard deviation

MD, MR-dominant; MLCD, MR-LV-co-dominant; MI, myocardial infarction; CABG, coronary artery bypass grafting; PCI, percutaneous coronary intervention; CKD, chronic kidney disease; STS, Society of Thoracic Surgeons; BNP, brain natriuretic peptide; eGFR, estimated Glomerular Filtration Rate ; ACEi, angiotensin converting enzyme inhibitor; ARB, angiotensin II receptor blocker; ARNI, angiotensin receptor neprilysin inhibitor; MRA, mineralocorticoid receptor antagonist; MR, mitral regurgitation; LVEF, left ventricular ejection fraction; LAD, left atrial diameter; LVDd, left ventricular end-diastolic diameter; LVDs, left ventricular end-systolic diameter; LVEDV, left ventricular end-diastolic volume; EROA, effective regurgitant orifice area; PISA, proximal isovelocity surface area

**Table 2:** 1-year postprocedural patient characteristics

	MD/MLCD
Patients, n	*n* = 17
Postprocedural variables	
Laboratory data	
BNP, pg/mL	306.7 ± 309.2
eGFR, mL/min/1.73 m^2^	39.7 ± 17.1
Hemoglobin, mg/dL	12.6 ± 1.5
Medications	
ACEi/ARB/ARNI, n	11 (64.7)
βblocker, n	16 (94.1)
MRA, n	11 (64.7)
Echocardiographic data	
MR grade, n	
I / II	17 (100.0)
LVEF, %	32.2 ± 12.2
LVDd, mm	60.1 ± 5.8
LVDs, mm	48.5 ± 8.4
LAD, mm	44.2 ± 14.8

Values are expressed as number (%) or mean ± standard deviation

MD, MR-dominant; MLCD, MR-LV-co-dominant; MR, mitral regurgitation; BNP, brain natriuretic peptide; eGFR, estimated Glomerular Filtration Rate; ACEi, angiotensin converting enzyme inhibitor; ARB, angiotensin II receptor blocker; ARNI, angiotensin receptor neprilysin inhibitor; MRA, mineralocorticoid receptor antagonist; LVEF, left ventricular ejection fraction; LAD, left atrial diameter; LVDd, left ventricular end-diastolic diameter; LVDs, left ventricular end-systolic diameter

#### The changes of MIBG scintigraphy parameters

**Fig. 2:**
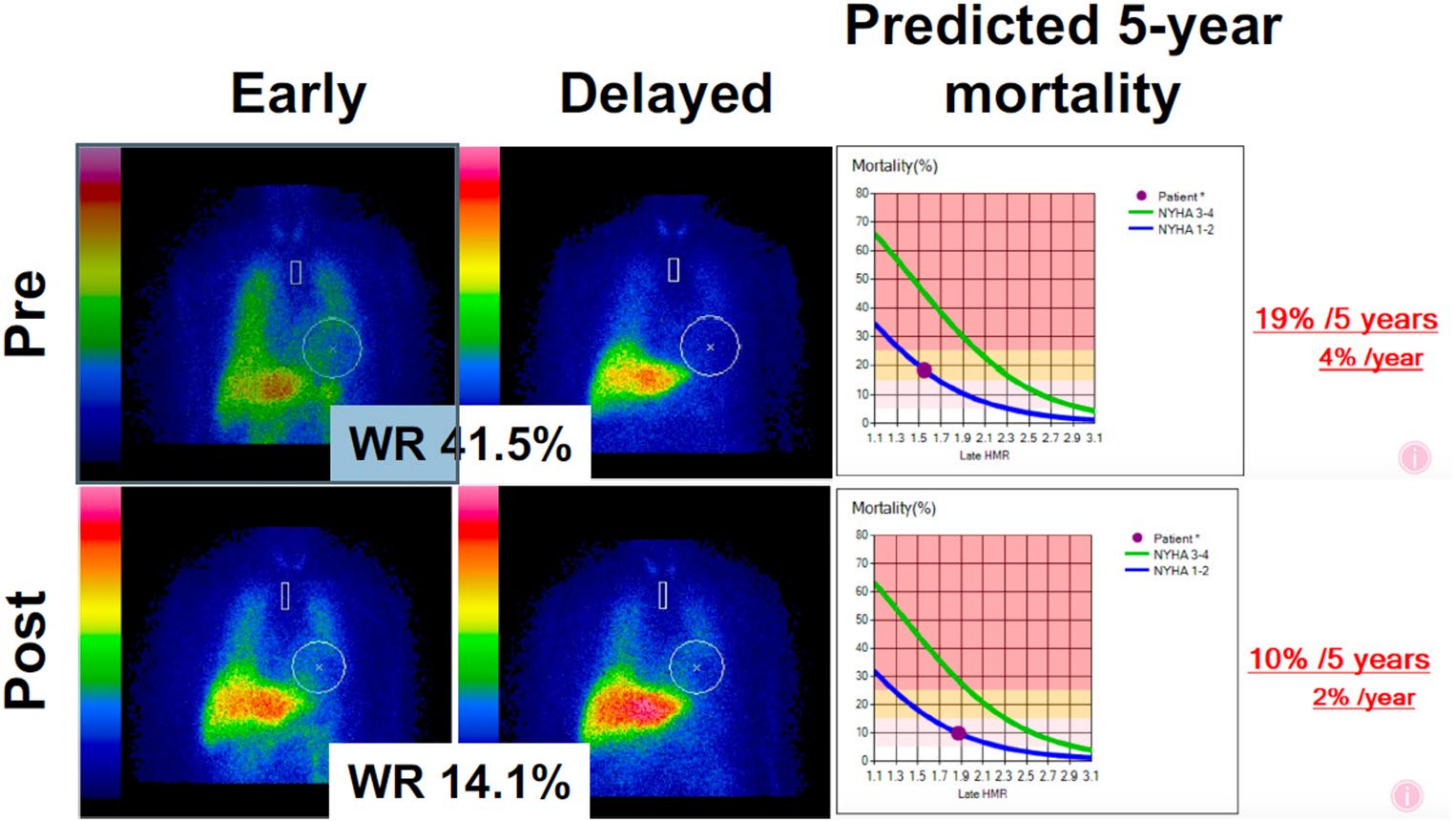
Representative case of MD/MLCD group

**Fig. 3:**
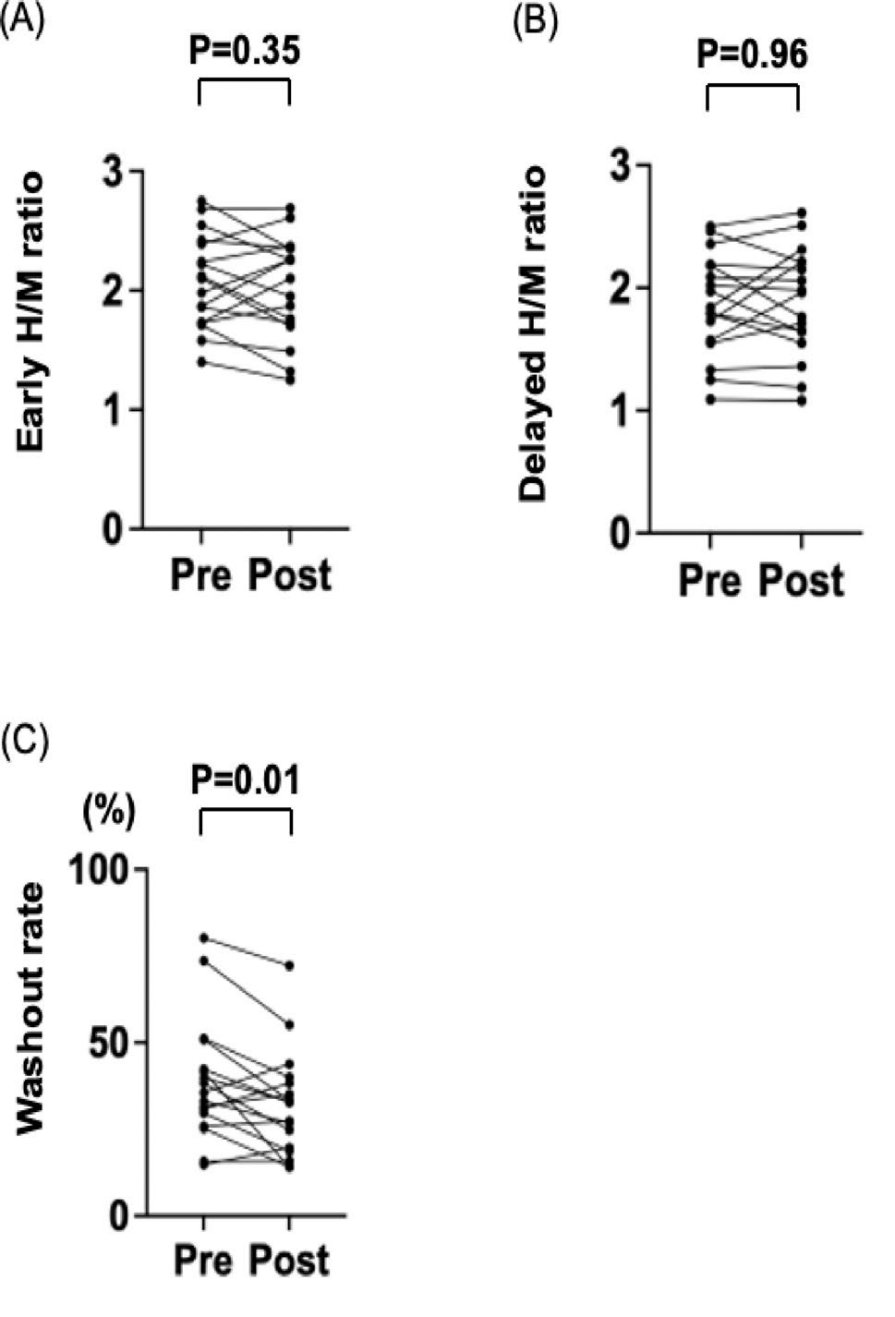
**A** Change in e-H/M post-TEER. **B** Change in d-H/M post-TEER. **C** Change in WR post-TEER

**Fig. 4:**
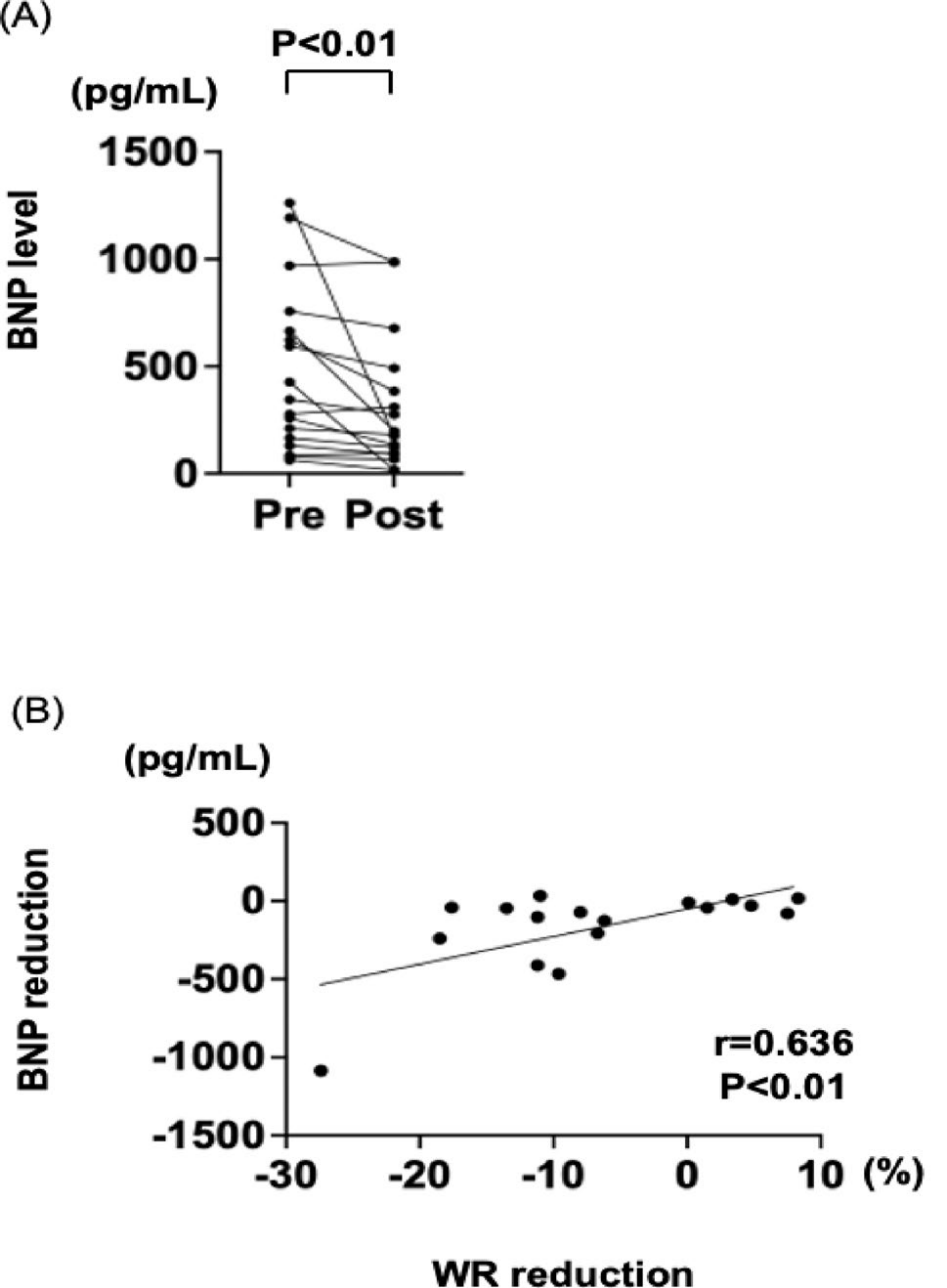
**A** Change in BNP level post-TEER. **B** The correlation between WR and BNP decreases

The median interval from baseline MIBG to M-TEER was 2 days (IQR 1.5-8.0). The median interval from M-TEER to follow-up MIBG was 356 days (IQR 341-363). Representative case of MD/MLCD group was shown in Fig. 2. In terms of MIBG parameters, e-H/M and d-H/M showed no significant changes (e-H/M; 2.07 ± 0.39 vs. 2.01 ± 0.43, *P* = 0.35, d-H/M; 1.86 ± 0.41 vs. 1.87 ± 0.44, *P* = 0.96) (Fig. 3A, B). On the contrary, WR significantly decreased post-M-TEER from 38.8 ± 17.5 % to 32.1 ± 15.2 %, with a mean change of – 6.7 % (95% CI–11.8 to –1.6, *P* = 0.01) (Fig. 3C). The BNP level was significantly decreased post-M-TEER from 476.7 ± 387.1 pg/mL to 306.7 ± 309.2 pg/mL, with a mean change of –169.9 pg/mL (95% CI –311.7 to –28.1, P < 0.01) (Fig. 4A). Decrease in WR was significantly correlated with BNP decrease (r = 0.636, P < 0.01) (Fig. 4B). Model-based 5-year mortality risk was unchanged post-M-TEER (25.9 ± 15.7 % vs. 20.4 ± 11.3 %, *P* = 0.45).

## Discussion

### Main findings

This study identified a significant reduction in CSN tone, as indicated by a decrease in WR, among MD/MLCD patients who underwent M-TEER. To our knowledge, this is the first report demonstrating a reduction in CSN tone following M-TEER, as verified by MIBG scintigraphy among disproportionate SMR patients.

### CSN activity assessed by MIBG and M-TEER

This study revealed WR improved without significant changes in e-H/M or d-H/M. Dysfunction in cardiac sympathetic nerve activity is associated with poor outcomes in patients with heart failure. MIBG, an analog of norepinephrine, is a useful tool for detecting CSN abnormalities in HF patients [15]. Among MIBG parameters, e-H/M primarily reflects the density of presynaptic sympathetic nerve terminals and uptake-1 function, whereas WR reflects sympathetic drive, and d-H/M integrates both structural and functional components. Among these parameters, d-H/M is the most validated prognostic parameter in large prospective studies such as ADMIRE-HF [16]. Reduced d-H/M and higher WR are reported to be associated with worse prognosis in heart failure [3, 4, 17]. 

The finding that WR improved without accompanying changes in either e-H/M* or d-H/M* indicates attenuation of sympathetic tone rather than restoration of sympathetic innervation. Candidates for M-TEER were required to receive maximally optimized guideline-directed medical therapy (GDMT) prior to the procedure, and medical therapy is known to influence MIBG parameters. Among heart failure therapies, β-blockers have been shown to improve LVEF and reduce LV dimensions, leading to increased H/M 6 months after initiation [18]. Mineralocorticoid receptor antagonists similarly improve LVEF and reduce LV dimensions, resulting in improvements in both H/M and WR [2]. Sodium-dependent glucose transporter 2 inhibitors improve LVEF without altering LV dimensions within 3 months, and neither H/M nor WR changes significantly within this period [19]. Taken together, these findings suggest that long-term LV improvement and reverse remodeling induced by medical therapy for heart failure could contribute to improved MIBG parameters. In the present study, all patients were already treated with maximally optimized GDMT before M-TEER, which was maintained without substantial adjustment following the intervention. Thus, the therapeutic benefits of GDMT had already been realized before the procedure. The role of M-TEER is to provide an additive effect beyond GDMT, thereby improving clinical outcomes. Although M-TEER for SMR may not recover LVEF, it can increase cardiac output [20–22], which may account for the improvement in WR without significant alterations in either e-H/M or d-H/M. M-TEER may mitigate sympathetic overactivation, despite its limited capacity to restore sympathetic neural integrity.

### MIBG findings and BNP

We observed significant reductions in both WR and BNP following M-TEER in patients with disproportionate MR. Moreover, the reduction in WR was significantly correlated with the decline in BNP. BNP reflects hemodynamic burden, wall stress, volume overload, and filling pressures, yet it is also influenced by several non-cardiac factors—including renal function, age, body composition, and acute clinical fluctuations [23, 24]. In contrast, MIBG imaging provides a direct and quantitative assessment of cardiac sympathetic nerve activity, a key determinant of prognosis. Among MIBG parameters, d-H/M has been shown in large prospective cohorts to hold independent prognostic value beyond natriuretic peptides, even in patients with elevated BNP levels [16]. MIBG provides incremental prognostic value in patients with higher BNP levels, suggesting that it is also informative in individuals with more advanced heart failure [4, 16].

### MIBG change and proportionality

This study identified a significant reduction in CSN tone, as indicated by a decrease in WR among disproportionate MR. Previous studies suggest that M-TEER enhances CSN tone. [6]. Another study demonstrated that M-TEER improves CSN tone in primary MR (PMR) patients, whereas it does not yield similar benefits in SMR patients [25]. The reason may arise from the fact that PMR primarily involves leaflet pathology, while SMR encompasses not only mitral valve complex issues but also left ventricular (LV) dysfunction. In PMR, addressing leaflet issues through M-TEER may facilitate improvements in CSN tone. Conversely, in SMR patients, resolving complexities of the mitral valve may be insufficient, as LV issues persist. In heart failure with reduced ejection fraction, increased sympathetic nervous system responses compensate for diminished cardiac output [26]. If LV problems remain unaddressed, CSN tone is unlikely to improve. This study identified a significant reduction in CSN tone, as indicated by a decrease in WR among disproportionate MR. Because disproportionate MR is predominantly valve-driven, it more closely resembles PMR within the spectrum of SMR. Accordingly, our findings appear consistent with previous observations.

Conflicting results from *MITRA-FR (Percutaneous* Repair with the MitraClip Device for Severe Functional/Secondary Mitral Regurgitation) [27] and *COAPT (Cardiovascular* Outcomes Assessment of the MitraClip Percutaneous Therapy for Heart Failure Patients with Functional Mitral Regurgitation) [5] may be explained by the concept of proportionality, defined by the EROA/LVEDV ratio. Proportionate MR is driven predominantly by LV dilation, while disproportionate MR reflects more severe MR relative to LV size and is more valve-driven. Patients with disproportionate MR—more common in COAPT—may benefit more from M-TEER.

### Outcomes and proportionality

Evidence linking proportionality to outcomes after M-TEER remains inconsistent: some studies showed better survival in patients with disproportionate MR, while others found no prognostic association [8, 28]. These discrepancies may reflect the concept’s recent introduction and lack of a standardized definition. Further randomized studies are needed to clarify whether proportionality predicts post-M-TEER outcomes.

Reduced CSN tone may contribute to improved outcomes post-M-TEER in patients with disproportionate MR. However, the 5-year predicted mortality model did not demonstrate superior outcomes. The 5-year predicted mortality risk was automatically calculated using smartMIBG^Ⓡ^, which integrates clinical data—including age, sex, and NYHA class—with MIBG parameters [13, 14]. One potential explanation is that other prognostic factors, such as renal function and anemia, also influence outcomes after M-TEER, but these variables are not accounted for in the 5-year predicted mortality risk calculation.

The divergent WR trajectories in disproportionate MR patients may serve as an early prognostic marker following M-TEER. The reduction of WR suggests enhanced autonomic function and improved cardiac stability. However, the absence of statistical significance in the predicted 5-year mortality risk, which is a model-based prediction, observed in this study likely reflects the small sample size and the multifactorial nature of long-term prognosis. While WR changes provide valuable insights into autonomic regulation, additional factors such as renal function, anemia, and comorbidities likely influence survival outcomes, which are not fully accounted for by the model. If validated in larger cohorts, post-M-TEER WR changes could emerge as a crucial predictor of long-term outcomes.

### Study limitations

This study has several limitations that should be acknowledged. First, patients without baseline or follow-up MIBG scintigraphy data were excluded, resulting in a limited sample size. Furthermore, the exclusion of these patients may have led to selection bias. Further studies with larger cohorts are necessary to validate our findings. Second, the follow-up duration was relatively short. Long-term follow-up is required to establish the relationship between prognosis after M-TEER and the improvement in CSN activity. Third, the variability in the intervals between baseline MIBG and M-TEER, as well as between M-TEER and follow-up MIBG, may have influenced the results. Finally, CSN improvement may be attributable not only to M-TEER but also to concurrent medical treatments. A comparison of CSN activity between M-TEER plus medical treatment and medical treatment alone is necessary to confirm the specific effect of M-TEER.

## Conclusion

This study observed a significant reduction in CSN tone, as indicated by a decrease in WR, among disproportionate patients who underwent M-TEER.

## Abbreviations

M-TEER: Mitral transcatheter edge-to-edge repair
SMR: Secondary mitral regurgitation
CSN: Cardiac sympathetic nerve
MIBG: ^123^I-metaiodobenzylguanidine
EROA: Effective regurgitant orifice area
LVEDV: Left ventricular end-diastolic volume
WR: Washout rate
H/M: Heart-to-mediastinum ratio
MD/MLCD: Mitral regurgitation-dominant/ mitral regurgitation-left ventricular-co-dominant
LD: Left ventricular-dominant
BNP: Brain natriuretic peptide
